# Whole-genome duplication and molecular evolution in *Cornus* L. (Cornaceae) – Insights from transcriptome sequences

**DOI:** 10.1371/journal.pone.0171361

**Published:** 2017-02-22

**Authors:** Yan Yu, Qiuyun Xiang, Paul S. Manos, Douglas E. Soltis, Pamela S. Soltis, Bao-Hua Song, Shifeng Cheng, Xin Liu, Gane Wong

**Affiliations:** 1 Department of Plant and Microbial Biology, North Carolina State University, Raleigh, NC, United States of America; 2 Key Laboratory of Bio-Resources and Eco-Environment of Ministry of Education, College of Life Sciences, Sichuan University, Chengdu, Sichuan, PR China; 3 Department of Biology, Duke University, 130 Science Drive, Durham, NC, United States of America; 4 Florida Natural History Museum, University of Florida, Gainesville, FL, United States of America; 5 Department of Biology, University of Florida, Gainesville, FL, United States of America; 6 Department of Biological Sciences, University of North Carolina at Charlotte, 9201 University City Blvd, Charlotte, NC, United States of America; 7 BGI-Shenzhen, Shenzhen, China; 8 Department of Biological Sciences and Department of Medicine, University of Alberta, Edmonton, Alberta, Canada; National Cheng Kung University, TAIWAN

## Abstract

The pattern and rate of genome evolution have profound consequences in organismal evolution. Whole-genome duplication (WGD), or polyploidy, has been recognized as an important evolutionary mechanism of plant diversification. However, in non-model plants the molecular signals of genome duplications have remained largely unexplored. High-throughput transcriptome data from next-generation sequencing have set the stage for novel investigations of genome evolution using new bioinformatic and methodological tools in a phylogenetic framework. Here we compare ten de novo-assembled transcriptomes representing the major lineages of the angiosperm genus *Cornus* (dogwood) and relevant outgroups using a customized pipeline for analyses. Using three distinct approaches, molecular dating of orthologous genes, analyses of the distribution of synonymous substitutions between paralogous genes, and examination of substitution rates through time, we detected a shared WGD event in the late Cretaceous across all taxa sampled. The inferred doubling event coincides temporally with the paleoclimatic changes associated with the initial divergence of the genus into three major lineages. Analyses also showed an acceleration of rates of molecular evolution after WGD. The highest rates of molecular evolution were observed in the transcriptome of the herbaceous lineage, *C*. *canadensis*, a species commonly found at higher latitudes, including the Arctic. Our study demonstrates the value of transcriptome data for understanding genome evolution in closely related species. The results suggest dramatic increase in sea surface temperature in the late Cretaceous may have contributed to the evolution and diversification of flowering plants.

## Introduction

Whole-genome duplication (WGD), or polyploidy, followed by gene loss and diploidization has long been recognized as an important evolutionary force in plants [1–4]. Although some have considered polyploids as evolutionary ‘dead-ends’ [5–7], abundant evidence supports that polyploidy, either by spontaneous doubling of chromosome sets in somatic cells or by union of unreduced gametes, is a major evolutionary mechanism of plant diversification [2, 8].

Among the limited number of seed plants for which complete nuclear genome sequences are available, all show evidence of one or more rounds of ancient polyploidization [9]. Comparative analyses suggest that the major radiations of seed plant clades, specifically the angiosperms [9] and the eudicots [10], were accompanied by WGDs. Data from whole-genome sequences of model plants (e.g. *Arabidopsis*) and agriculturally important species from the Asteraceae, Brassicaceae, Fabaceae, Poaceae, and Solanaceae also indicate multiple independent WGD events [11–13]. Furthermore, a recent analysis of 41 plant genomes by Vanneste et al. [14] revealed a strong, non-random pattern of genome duplications over time, with many WGDs clustered around the Cretaceous-Paleogene (K-Pg) boundary. Following WGD, different evolutionary processes act on the duplicated genes and direct their fates. For example, some duplicated gene copies might be retained by purifying selection, while others evolve into new genes with new functions under strong diversifying selection. The evolutionary processes driving molecular evolution can be revealed by the substitution rates at synonymous (*Ks*) and nonsynonymous (*Ka*) sites. A neutral evolutionary process maintains an approximately equal rate of *Ka* and *Ks* (*Ka/Ks* = 1), while purifying selection (*Ka/Ks*<1) eliminates nonsynonymous substitutions, and positive/diversifying selection (*Ka/Ks*>1) favors nonsynonymous substitutions. These processes may lead to various outcomes, such as pseudogenization, functional conservation, and divergence of the duplicated genes. Genome-wide increases of synonymous and nonsynonymous substitution rates following WGD are expected to drive paralogous gene copies to diverge. This pattern was demonstrated in *Arabidopsis* [15], maize [16], rice [17], and wheat [18]. In contrast, gene conversion via concerted evolution acts to homogenize paralogous gene copies, reducing the signals of gene divergence [19].

WGDs can be detected from an estimated age distribution of paralogous genes [9, 14, 20–22]. The age distribution of paralogs is commonly estimated through a distribution of values for the average number of synonymous substitutions per synonymous site (*Ks*) [22, 23]. Because synonymous substitutions do not alter protein products, they are putatively neutral and expected to accumulate at an approximately constant rate [24]. The peaks of *Ks* values clearly indicate that a massive set of paralogs is one consequence of WGD, and the timing of that peak can be estimated using a molecular clock. Although *Ks*-based methods do not require collinear gene data [25], they may be affected by the accuracy of inferred *Ks* values, which are prone to saturation effects. *Ks*-based methods are considered to be most appropriate to detect recent WGDs with *Ks* values of less than 2 [26]. Another limitation of this approach is potential error of the molecular clock used to convert the *Ks* values to time. Applying an “universal” clock across lineages can be significantly biased. An alternative approach uses the frequency distribution of dates of gene duplication events shown on gene trees to assess WGD [9]. This approach is more reliable if the dating analysis of the gene trees can be appropriately done with fossil calibrations. Nonetheless, the *Ks* approach has been used to identify recent as well as ancient genome duplications in plants with transcriptome data [21, 22]. For example, the WGD(s) in the ancestor of the core eudicot clade has been independently identified [10, 14, 26–29]. This WGD is sometimes referred to as an ancient ‘hexaploidization’ but mechanistically it would have originated as two duplication events in close temporal succession.

Few studies have explored the relationships among genome duplication, molecular evolution, and the pattern of species diversification. High-throughput transcriptome data obtained using next-generation sequencing technology and the development of related analytical tools have enabled novel investigations of genome evolution through comparative analyses in closely related species [30–32]. Evidence from additional plant lineages occupying important phylogenetic positions will be particularly helpful to improve our understanding of genome evolution in angiosperms.

*Cornus* L. (dogwood) is a member of Cornales, the sister group to all other asterids [33, 34], and consists of four morphologically distinct major clades [35, 36]. These are the blue- or white-fruited dogwoods (BW), the cornelian cherries (CC), the big-bracted dogwoods (BB), and the dwarf dogwoods (DW). Previous studies revealed rapid divergence of BW, CC, and BB-DW at the boundary of the late Cretaceous and Paleocene and subsequent divergence within each major clade in the early Tertiary before 40 myrs ago [35, 37]. *Cornus* thus provides an ideal system in which to assess whether WGD and increased molecular evolution were associated with the early diversification of the genus. If so, we expect to find evidence of WGD events dating back to the late Cretaceous in all *Cornus* species, prior to, but close to the time of the early diversification of the genus. *Cornus* also has a rich fossil record [38–40], making it an excellent group for comparing the *Ks* and “gene tree” method in identifying and dating WDG events with transcriptome data.

In this study, we analyze ten *de novo*-assembled transcriptomes from eight species of four major lineages of *Cornus* and outgroups to address the following questions: 1) When and where in the phylogeny did WGD likely occur? 2) What is the pattern of rate changes in molecular evolution following WGD? 3) How do the rates of molecular evolution vary among lineages through time? And 4) Are results from the *Ks* method congruent with those from the “Gene tree” method?

## Material and methods

### Transcriptome data

Total RNA was extracted using a modified CTAB RNA isolation method [41]. The cDNAs were first synthesized using Evrogen’s (Moscow, Russia) MINT Universal cDNA synthesis kit (cat# SK005) and then normalized using the Evrogen Trimmer kit (cat# NK003). The normalized libraries of *Cornus elliptica* (CEL) and *Cornus kousa* (CKO) were processed into sequencing libraries using Roche’s standard Rapid Library kit (cat# 05 608 228 001; all procedures following manufacturer’s recommendations).

Raw transcriptome data were obtained as part of a coordinated series of research efforts. Sequences of *Cornus canadensis* (CCN) were obtained from a previous study that used 454 sequencing of inflorescence and leaf samples (a non-normalized library) [42]. Sequences of the outgroups, *Alangium chinense* (ACH) and *Dichroa febrifuga* (DFE), were generated by Beijing Genomics Institute (BGI) with 90 bp paired end sequencing on Illumina HiSeq 2000 and made available courtesy of the One Thousand Plants (1KP) Transcriptome Project (http://www.onekp.com). [43–45]

Transcriptome data for seven *Cornus* species were newly generated for this study at North Carolina State University (NCSU Genomic Science Lab) and BGI. For *Cornus kousa* and *C*. *elliptica*, normalized libraries were made using a TruSeq RNA library prep with mean size of 366 bp and 362 bp, respectively, and run for 72- bp paired-end sequencing on a GAIIx Illumina sequencer at the Genomic Science Lab at NCSU. For *Cornus alternifolia* (CAL), *C*. *capitata* (CCA), *C*. *controversa* (CON), *C*. *florida* (CFL), and *C*. *officinalis* (COF), non-normalized libraries were run for 150 bp paired-end sequencing on the Illumina HiSeq-2000 platform. Leaf material was collected from living plants grown locally in the J.C. Raulston Arboretum or the Sarah P. Duke Garden with the permission and assistance of director Mark Weathington, (mweathi@ncsu.edu) and curator Paul Jones (pdjones@duke.edu), respectively.

Assembly and analyses of the transcriptome data followed a customized pipeline illustrated in S1 Fig to ensure quality assembly, reliable identification of orthologous and paralogous genes, and robust estimation of substitution rates and molecular dating (described below).

### Data trimming and de novo assembly

The raw reads of *C*. *alternifolia*, *C*. *capitata*, *C*. *controversa*, *C*. *elliptica*, *C*. *florida*, *C*. *kousa*, *C*. *officinalis*, *A*. *chinense*, and *D*. *febrifuga* were trimmed at the 3’ end when the Phred quality score of a read dropped below Q = 20 (or 0.01 probability of error) for two consecutive bases. All 5’ and 3’ stretches of ambiguous ‘N’ nucleotides and sequences of less than 20 bp were removed from sequence trimming using CLC Genomics Workbench 4.6.1 (CLC Bio, Aarhus, Denmark). Similarly, the low-quality sequences, ambiguous nucleotides, adapter sequences, short sequences (<20 bp), and 454 sequence primers were removed from the raw reads of *C*. *canadensis* through data trimming. The filtered high-quality reads were then analyzed using a customized pipeline of software and methods (S1 Fig) as described below. The high-quality reads were then *de novo*-assembled using Trinity 2.1.3 [46], and the minimum sequence length in the assembly was set to 300 bp. The isoforms from the final output were treated as unique sequences, although each contig may contain several isoforms. FASTQ sequence files for each taxon have been deposited in the Sequence Read Archive (SRA) database at NCBI (SRP072429).

### Identification of orthologous and paralogous genes for Ks, Ka, and Ka/Ks estimation

We used a slightly modified version of the *Ks*-based method of Jiao et al. [9] and McKain et al. [27] to analyze the transcriptome sequence data for the signal of one or more ancient genome duplications across all taxa. Our method differed mainly by converting the *Ks* values into absolute ages using the dated single-copy gene tree of *Cornus*. All-by-all BLASTN searches were performed on the combined transcriptome sequences of all ten species with an e-value cutoff of 10^−6^ and identity of at least 40%. The paralogous and orthologous pairs were then identified as best matches within and between species, respectively. To remove possible redundancy in the transcriptome sequence data, the paralogs that belong to the same contig (gene) were excluded from further analysis [27]. If more than one paralog/ortholog pairs were detected between any two genes, only the pair with the longest alignment length was retained. Amino acid sequences were estimated for these homologs using the program ESTscan 2.0 [47]. Paralog and putative ortholog matches with minimum alignment lengths of 150 bp and at least 60% identity were analyzed further. These cutoffs were used to provide a minimum of 50 codons for alignments used in the estimation of the number of synonymous substitutions per synonymous site. The paired orthologs or paralogs with no gaps were exported using in-house Perl scripts (available in http://mnh.scu.edu.cn/perl). Pairwise *Ks*, *Ka*, and *Ka/Ks* values of each ortholog and paralog pair were then calculated using codeml in the PAML 4 package) [48], using paired sequence settings (yn00) [49] and the F3by4 model [50] for estimating codon frequencies needed for calculating *Ka* and *Ks*.

### Estimating phylogeny and divergence times of putative orthologs

We reconstructed a calibrated phylogeny of the *Cornus* species under study and rooted the tree using *Dichroa* (Hydrangeaceae, a close relative of Cornaceae). We used putative single-copy genes (SCG) to build the phylogeny to serve as our framework for investigating the temporal patterns of molecular evolution and genome duplication. To identify genes that are single-copy in all ten species, orthologous groups were first predicted using OrthoMCL v2.0 [51] with default settings. Orthologous groups containing only one sequence variant from each species were treated as SCG groups (thus, excluding genes with allelic variation within a species); and each group was aligned using MUSCLE v3.7 [52].

The Bayesian Inference (BI) tree for each SCG group was estimated by MrBayes v3.2.2 [53]. Two independent MCMC analyses were run, each with one cold chain and three heated chains. Each chain was run for at least 10 million generations (λ = 0.2), sampling trees every 1000 generations with the first 25% of trees sampled discarded as burn-in. ESS (Effective Sample Size) was checked using Tracer v1.6 [54] to be sure that sufficient sampling occurred (ESS > 100). The tree topologies of each SCG were then checked for conflicting signals using Bucky v1.4.2 [55].

These SCGs were then concatenated into a supermatrix for divergence time estimation. The BI SCG trees with congruent tree topology were combined into a consensus tree to calculate the average branch length and then used as a starting tree for divergence time analysis using BEAST 1.8.2 [56] with the Yule Process [57, 58] for the tree prior and the uncorrelated lognormal (UCLD) relaxed clock model [59]. Selected fossils used in previous phylogenetic dating analyses of *Cornus* and Cornales were used to constrain six nodes of the tree (S2 Fig, N1 to N6) [35, 37]. Details of the dating analysis (e.g. constraints and priors) are provided in S1 Table.

The dating analysis was performed with two independent runs of 100 million generations each. Trees and divergence time estimates were sampled once every 10,000 generations, and the results of each run were checked for convergence with the software Tracer v1.6 [54]. The burn-in of each run (10–20%) was determined according to the plot of the tree likelihood scores. Tree samples after burn-in were combined from both runs and used to reconstruct the best tree using TreeAnnotator v.1.8.2 [56].

### Calibrating the local species clock for inferring the timing of WGD events from paralogous Ks

**Fig 1:**
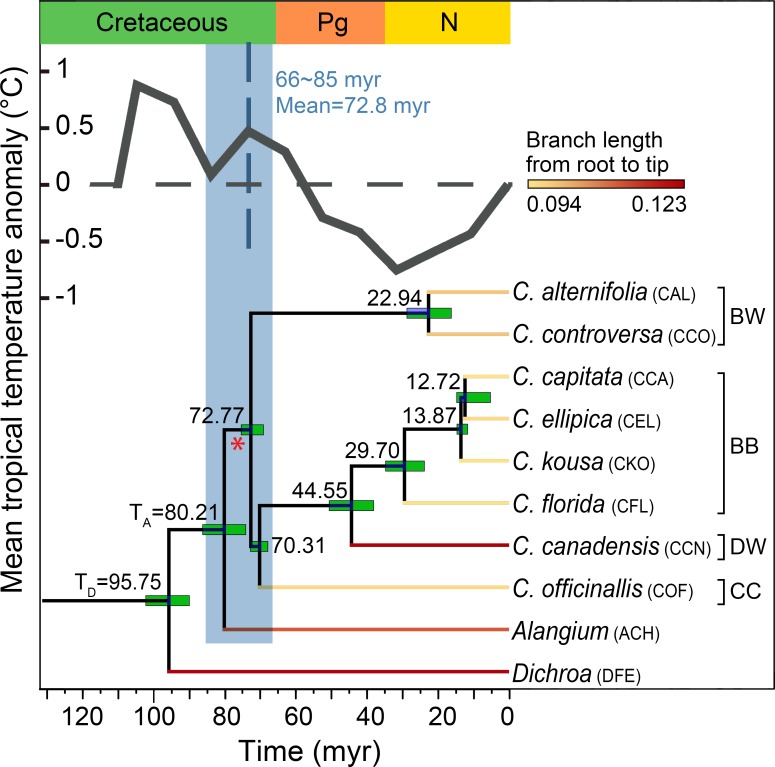
Phylogenetic reconstruction of the 10 study species using 51 single copy genes obtained from the transcriptome data. Three letter acronyms are used to refer to each major lineage. The tree was dated with fossil calibration points. The blue bar represents the range of estimated times for the WGD events detected in the *Cornus* species analyzed in this study. The location of WDG with the highest likelihood score from the hypothesis testing analysis is marked with an asterisk. The sea surface temperatures inferred from the d_18_O data were placed above the tree [82]. Estimated branch length is scaled by color intensity.

**Fig 2:**
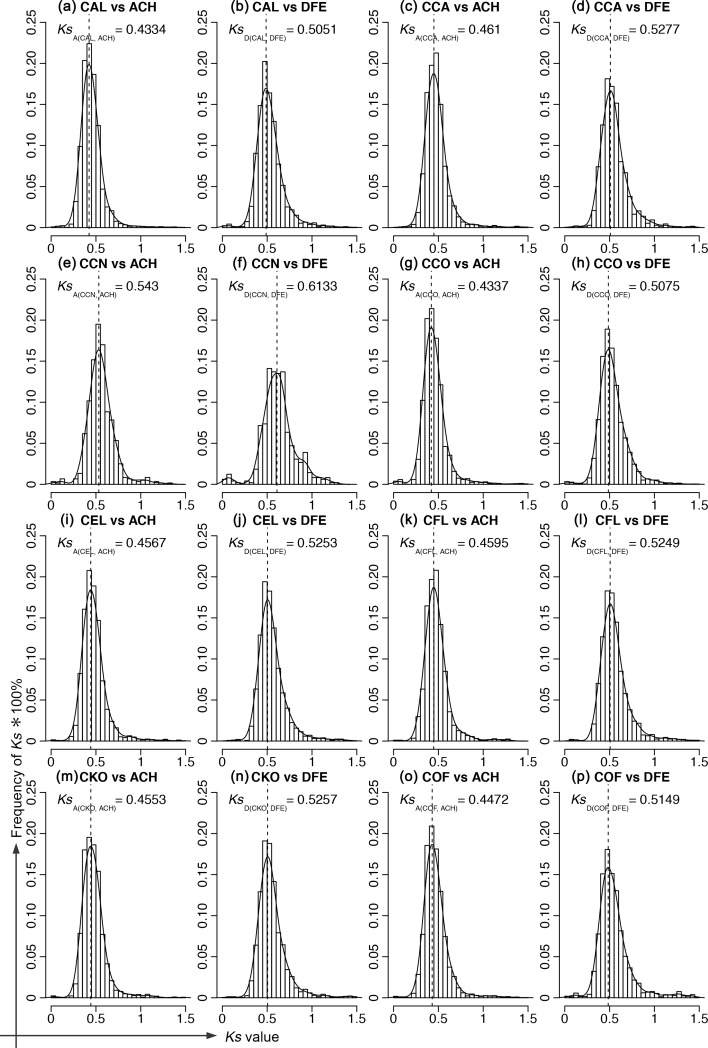
Distribution of synonymous substitutions *Ks* in orthologous pairs between species of *Cornus* and outgroups. The x-axix is the value of *Ks* and the y-axix is frequency of *Ks* *100%. The black curve represents the kernel density estimate of the distributions, while the vertical black dashed lines represent its peak (used as the molecular clock C_*Ks*_ for dating paralogous *Ks*) as inferred from the density function.

The rate of molecular evolution can vary widely among lineages and life-history strategies [60]. Assigning absolute ages to WGD events inferred from *Ks* frequency distribution can be difficult without a local clock for the relevant species [61]. Thus, converting *Ks* values to time for cross-species comparisons requires correction for rate variation among species. We used the *Ks* of putative orthologs to calibrate the clock with the age of divergence between *Cornus* and outgroups for dating WGDs. The clock (*C*_*Ks*_) for *Cornus* was calibrated through comparisons with the two outgroups (*Alangium* and *Dichora*) using two time points on the phylogeny, the age of the node uniting *Cornus* and *Alangium* (*T*_*A*_) and the age of the root [the node uniting *Cornus*, *Alangium*, and *Dichroa* (*T*_*D*_)] (Fig 1). The clock for a given *Cornus* species was estimated as the average of two calculations *Ks*_*A*_ / 2*T*_*A*_ and *Ks*_*D*_ / 2*T*_*D*_, where *Ks*_*A*_ was the synonymous substitution rate between putative orthologous genes in a given *Cornus* species and *Alangium*, and *Ks*_*D*_ was the synonymous substitution rate between orthologous genes in a given *Cornus* species and *Dichroa*. For instance, the clock of *C*. *alternifolia*, *C*_*Ks(CAL)*_ = (*Ks*_*A(CAL*,*ACH)*_ / 2*T*_*A*_ + *Ks*_*D(CAL*,*DFE)*_ / 2*T*_*D*_)/2; the clock of *C*. *canadensis*, *C*_*Ks(CCN)*_ = (*Ks*_*A(CCN*,*ACH)*_ / 2*T*_*A*_ + *Ks*_*D(CCN*,*DFE)*_ / 2*T*_*D*_)/2. *Ks*_*A*_ and *Ks*_*D*_ of each species of *Cornus* were determined as the value that corresponds to the peak of kernel density estimate of the *Ks* distribution from all orthologous pairs calculated using the density function [62] of the R package [63] with the automatic bandwidth selection (Fig 2).

The paralogous synonymous substitution rates were converted to absolute time of divergence by the equation *T* = *Ks*/2*C*_*Ks*_, where *T* is the absolute time of divergence, *Ks* is the paralogous synonymous substitution rate, and *C*_*Ks*_ is the molecular clock of the species. The frequencies of *Ks* values were plotted through time from 0 to 200 myr to identify WGD events (see below) and their relative timing.

### Modeling genome duplications

Mixture modeling techniques have proven successful in detecting even small deviations from mixed normal or gamma distributions [26]. To explore the distribution of *Ks* for signals of WGD events, the distribution of *Ks* was fit to a mixture model comprising several component distributions in various proportions. The multivariate normal components model was applied to the *Ks* frequency distributions through time within a 200 myr time frame using the mixture model test implemented in the program EMMIX [64]. Only genes with two or more pairs of paralogs were included in the analysis. The distribution of *Ks* values of paralogous pairs was modeled with two to five groups. The EM algorithm was repeated 100 times with random starting values, as well as 100 times with k-mean starting values. The best mixture model was identified using the Bayesian information criterion [9], and only groups including > 20% of the total number of paralog pairs were retained. The estimated mean (and standard deviation) for each group was then converted into absolute time using the clock of the corresponding species to infer the timing of a putative WGD. The absolute time of all *Cornus* species were then combined into one dataset and the mixture model test was applied to see if the combined data revealed similar pattern of *Ks* distribution as seen in the data from individual species. To explore the pattern of molecular evolution following WDG events, we also examined the distribution of *Ka/Ks* calculated with codeml [48] through time from 200 myr to the present in 5 myr increments for each *Cornus* species and the median value of *Ka/Ks* of combined data from all species. All calculations and figures were made in the R package [63].

### Identifying and dating WGD events using gene trees

To determine where on the phylogeny and how many times the WGD events have occurred, we considered five hypotheses (S2 Fig, H1 –H5). H1: WGD on the stem of the *Cornus-Alangium* clade; H2: WGD on the stem of the *Cornus* clade; H3: independent WGDs on the stem of the *Cornus* clade and on the stem of *Alangium*, respectively; H4: independent WGDs on the stem of the *Cornus* BW group (represented by CAL and CCO on S2 Fig) and on the stem of the remainder of the genus (represented by CCA, CEL, CKO, CFL, CCN and COF on S2 Fig), respectively; and H5: independent WGDs on the stem of *Alangium*, on the stem of the *Cornus* BW group, and on the stem of the remainder of the genus.

We tested the five hypotheses by comparing their likelihood scores using the R package WGDgc 1.2 [25] following the manual with the retention rates of WGDs from 0 to 1.0 steps 0.1 using the gene count data. WGDgc uses gene count data (number of gene copies) across multiple gene families from the results of OrthoMCL. The background process of gene duplications and gene losses is modeled by a birth and death process [25]. In the analysis, an orthologous gene group present in at least two species of *Cornus* and one of the outgroups was treated as a multiple gene group in each species. The multiple gene groups were then converted into gene count data using Excel (Microsoft 2010).

For comparison with the results based on the use of the *Ks* data, gene trees of orthologous groups were built and dated to infer gene duplication events. The orthologous groups containing at least two copies in a species from any of the four major clades of *Cornus* and at least one copy in *Alangium* and/or *Dichroa* were treated as groups with evidence for gene duplication (S3 Fig). Aligned sequences of each gene duplication group were generated using MUSCLE [52] set to default parameters. Maximum likelihood analyses were conducted using RAXML v8 [65] with the GTRGAMMA model. The sequence of the more distantly related outgroup *Dichroa* was removed manually from trees when its branch (from tip to root) was two times longer than other species (due to missing data or other reasons) to avoid spurious estimates of branch length in divergence time estimation.

Dating the gene trees requires time calibration of at least one node. Using the earliest fossil record of *Cornus* (late Cretaceous CC-like fruit from India and Vancouver Island; Manchester et al. in prep [66, 67]), we performed divergence time analyses using gene trees with a constraint of ≥68 million years for the *Cornus* crown node represented by the two lineages from the initial divergence of the genus. As transcriptome data may not capture all gene copies in every species (e.g. due to non- or low-expression of a gene copy or sequencing error), some orthologous gene trees in our data may not contain the calibration node and the phylogenetic position for every gene duplication event. We therefore identified gene trees showing gene duplication in the common ancestor of *Cornus* and dated trees that contained the following components (S3 Fig): *Alangium* and two subclades of *Cornus*, each consisting of any two or more species that represent the deepest phylogenetic divergence within *Cornus* (one or more species from the BW group, i.e., *C*. *alternifolia* or *C*. *controversa* or both, and one or more species from the sister clade of BW, i.e., any of the remaining *Cornus* species included this study). Aligned sequences and phylogenetic trees are available in supplementary material. The divergence times of these gene trees and the node uniting the two paralogous clades were estimated under the assumption of a relaxed molecular clock using a truncated Newton optimization algorithm as implemented in the program R8S [68]. The two nodes indicating WGD gene duplication shared by all trees were constrained with a minimum age of 68 myr by the aforementioned fossil (S1 Table), and the node uniting *Cornus* and *Alangium* was constrained for a maximum age of 86.28 myr (the upper bound of divergence time of the node estimated from the SCG tree dating analysis, as described above) (S3A Fig). Other parts of the gene tree will vary with the species sampling in each the two *Cornus* subclades. We applied constraints to the node uniting BB, DW and CC with a minimum age of 58 myr, the node uniting BB and DW with a minimum age of 32 myr, and the node uniting BB species with a minimum age of 5.1 myr, if present (S3B Fig). These nodes in each gene tree also were constrained using fossils (S1 Table). The dating analyses were performed for each gene tree. The dates of gene duplications for the ancestral node of *Cornus* from these gene trees were then tested with mixture model for a normal distribution using EMMIX.

## Results

### Sequencing and assembly

The Illumina sequencing of seven *Cornus* species and two outgroups yielded 8,677,735 to 14,856,278 high-quality reads. Raw reads of *C*. *canadensis* from our previous 454 sequencing study [32] yielded 337,382 reads. *De novo* assembly of the reads for all ten species studied here were assembled into 15,704 to 157,608 unigenes, with an average length from 463 bp (*D*. *febrifuga*) to 741 bp (*C*. *alternifolia*) (Table 1).

**Table 1:** Summary of assembly for transcriptomes of ten species.

	*C*. *alternifolia*	*C*. *capitata*	*C*. *canadensis*	*C*. *controversa*	*C*. *ellipica*	*C*. *florida*	*C*. *kousa*	*C*. *officinallis*	*A*. *chinense*	D. febrifuga
HQ reads	13758984	11286133	337382	13888929	12520007	13409925	14856278	14162118	11867628	8677735
Total trinity genes	103765	117013	15704	129625	150157	132273	132273	157608	63456	94654
Total trinity transcripts	124564	141336	16406	150813	180999	146927	146927	190891	78289	111063
Average length	740.76	692.78	672.06	685.54	661.71	627.56	642.07	621.29	551.03	462.99
Percent GC	41.04	40.95	43.58	40.94	40.43	43.09	39.97	40.41	42.01	41.1
Branch Length ^a^	0.097	0.094	0.123	0.097	0.094	0.095	0.094	0.095	0.109	0.122

^a^ The branch lengths measured in the number of substitutions per site.

### Single-copy genes, genealogy, and divergence times

Analyses from OrthoMCL [51] resulted in the identification of 51 single-copy genes (SCG) from 86,865 orthologous groups. In our study, the allelic variants in the *Cornus* species were identified as different genes. The small number of SCGs could be caused by the small size of transcriptome of *C*. *canadensis* generated from 454 Titanium sequencing. Analyses using Bucky v1.44 [55] identified a set of 38 SCGs that generated congruent phylogenies. When concatenated into a supermatrix, the 38 SCGs resulted in an alignment containing 37,775 base pairs. The tree reconstructed from these SCGs was consistent with previously published molecular phylogenies [35, 37, 69], showing the relationship of (*Dichroa*, (*Alangium*, ((*C*. *alternifolia*, *C*. *controversa*), (*C*. *officinalis*, (*C*. *canadensis*, (*C*. *florida*, (*C*. *kousa*, (*C*. *elliptica*, *C*. *capitata*)))))))) (Fig 1 and S2 Fig). The estimated branch length for each species from root to tip ranged from 0.094 to 0.123 (number of substitutions per site) (Table 1 and Fig 1). Results from divergence time dating showed that the crown node uniting all ten species on the SCG tree was 95.75 (90.00–102.22) myr. The divergence time for the split between *Alangium* and *Cornus* was 80.21 (74.19–86.28) myr, followed by the deepest splits in *Cornus* occurring from 70.33–72.77 myr (Fig 1).

### Synonymous substitution rates between putative orthologous and paralogous genes

Synonymous substitution rates (*Ks*_A_ *and Ks*_*D*_) of single copy orthologous pairs between each *Cornus* species and the outgroups, *Alangium* (ACH) or *Dichlora* (DFE), ranged from 0.463 to 0.689 substitutions per synonymous site (Fig 2 and Table 2). The synonymous substitution clocks of *Ks* (*C*_*Ks*_, substitutions per site per million years) that were calculated using these values and the nodal ages *T*_*A*_ and *T*_*D*_ (Fig 1) for the eight *Cornus* species ranged from 2.64E-3 to 3.27E-3. Variation in the total number of paralogous genes among species, from 3360 in *C*. *canadensis* to 78338 in *C*. *ellipica*, is likely a result of differences in source material, e.g., leaf, flower buds, sampling times and growing environments, and sequencing technologies. With a time frame of 0–200 myr, the maximum *Ks* values (synonymous substitutions per synonymous site) between paralogs ranged from 1.07 to 1.32. The number of paralogous genes within the time frame ranged from 177 to 3978, and these were used to produce the *Ks* frequency plots. The summary statistics for eight species of *Cornus* are presented in Table 2.

**Table 2:** Summary statistics of eight *Cornus* species.

	*C*. *alternifolia*	*C*. *capitata*	*C*. *canadensis*	*C*. *controversa*	*C*. *ellipica*	*C*. *florida*	*C*. *kousa*	*C*. *officinallis*
Clock of Ks (C_Ks_)	2.67E-03	2.81E-03	3.29E-03	2.68E-03	2.79E-03	2.80E-03	2.79E-03	2.74E-03
Maximum included Ks	1.07	1.13	1.32	1.07	1.12	1.12	1.12	1.10
Ks_A_	0.4334	0.4610	0.5430	0.4337	0.4567	0.4595	0.4553	0.4472
Ks_D_	0.5051	0.5227	0.6113	0.5075	0.5253	0.5249	0.5257	0.5149
Total number of paralogous	44981	63530	3360	48886	78338	42566	73733	74222
Include paralogous	2669	3786	177	2668	3978	2390	3411	3328

**Fig 3:**
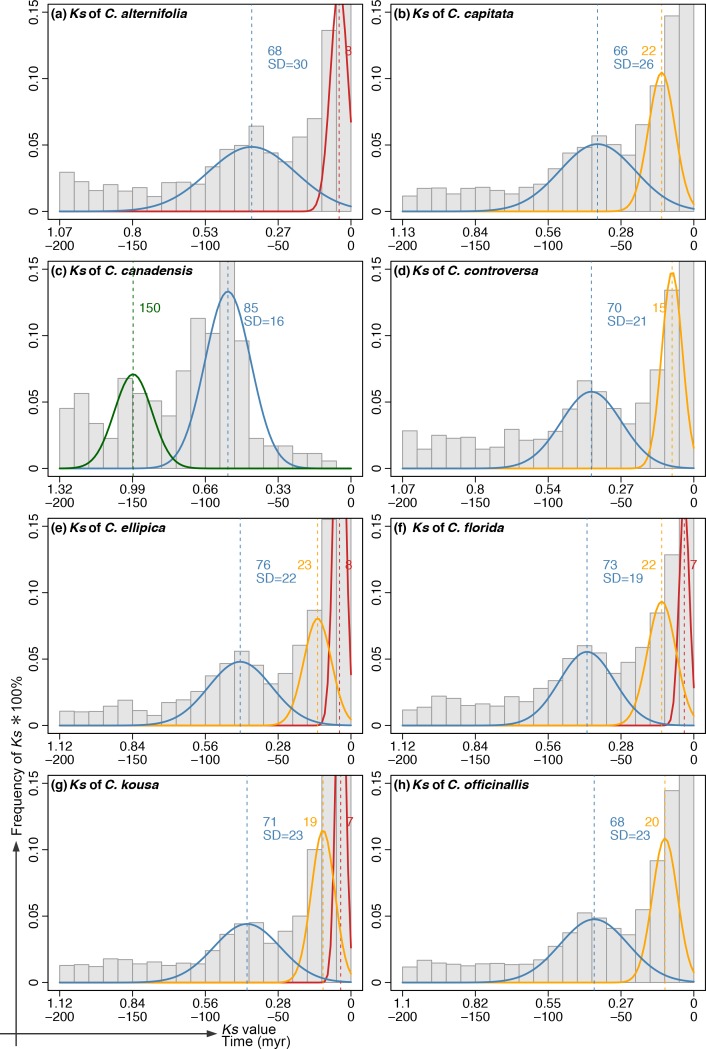
Frequency distribution of *Ks* values and corresponding ages of paralogous gene pairs in (a) *C*. *alternifolia*, (b) *C*. *capitata*, (c) *C*. *canadensis*, (d) *C*. *controversa*, (e) *C*. *ellipica*, (f) *C*. *florida*, (g) *C*. *kousa*, and (h) *C*. *officinallis*. Absolute ages were estimated using the *Ks* values with the molecular clock C_*Ks*_ calibrated for each species using outgroup references of *Alangium* and *Dichlora* (see Table 2). Normal distribution components of *Ks* were estimated using EMMIX (see Methods) and are superimposed on the histograms of the paralogous pair *Ks* plot (a-h). These components are hypothesized to be small-scale duplications (red and yellow curve), or whole genome duplications (green and blue yellow curve). The vertical green, blue, yellow and red dashed lines represent mean *Ks* values of the corresponding component (converted to absolute age). The estimated mean (and standard deviation) for each group was converted into absolute time using the clock of the corresponding species.

**Fig 4:**
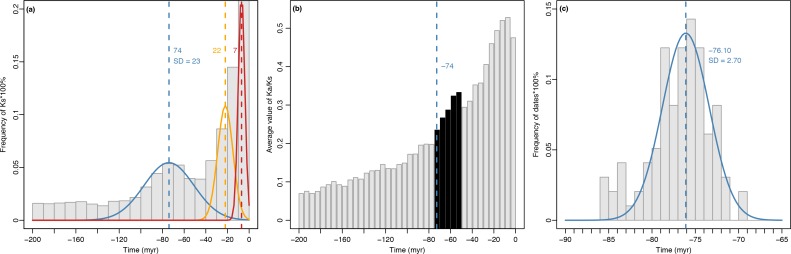
Frequency distributions of the ages and *Ka/Ks* values through time within a 200 million year window using combined data from all eight *Cornus* species and the frequency distributions of the dates of WGD in the *Cornus* ancestor inferred from gene tree method. a. The frequency distributions of the ages of paralogous genes. The normal distribution components of ages estimated using EMMIX and colored according to those in Fig 3. The vertical dashed lines represent average absolute ages of corresponding components (or WGD). b. *Ka/Ks* values through time within a 200 million year window. The grey bars represent the average values of *Ka/Ks* in a increment of 5 myr. and evident increase of *Ka/Ks* values after the inferred WGD event (marked by the blue vertical line and black bars). c. The frequency distributions of the dates of gene duplications at the *Cornus* ancestral node (inferred as the WGD event) from 98 gene trees. The normal distribution components of dates estimated using EMMIX. The vertical blue dashed line represent average dates of component.

Mixture model analyses revealed distinct components in the Ks frequency plots of each species that can be interpreted as small-scale duplications (SSDs) or WGDs. The Ks distributions of duplicates retained from SSDs are typically L-shaped, with abundant recent duplicates, but few old duplicates [26]. At issue is the obvious L-shaped background in the observed Ks distributions that presumably captured SSDs and loss across evolutionary time. Because Gaussian Mixture Modeling fits some Gaussian components in the rising part of this L-shape at lower Ks, it does not adequately fit the steep portion of the L-shaped distribution. Therefore, our interpretation is that the Gaussian components do not represent WGDs. The Bayesian information criterion (BIC) was used to choose the optimal number of normal distribution groups (NG) that fit the data for each *Ks* plot based on the EMMIX output. The BIC value of each NG (2–5) for each species (S2 Table), and detailed information is presented in S3 Table. Only groups including > 20% of the total number of paralog pairs were retained. The frequency distributions of *Ks* values with the estimated timing of WGD events for each species are shown in Fig 3. The *Ks* values for the peaks of the components observed from the *Ks* frequency plots of paralogous pairs are similar among the eight species and correspond to 150~159 myr (green lines), 66~85 myr (blue line), 15~23 myr (yellow line), and 7~8 myr (red line) (Fig 3). These peaks in the *Ks* distributions indicate the common timing of hypothesized WGDs or SSDs. For *Ks* varied in the rate among species, we converted the *Ks* into absolute time before combining them. The analyses from combined data of eight species of *Cornus* identified three major peaks (Fig 4A) with the mean values of 74 myr, 22 myr, and 7 myr, respectively (Fig 4A). The distributions of *Ka/Ks* values over time showed a similar pattern among species (S4 Fig). Increased *Ka/Ks* ratio after the late Cretaceous WGD event (66~85 myr, blue line in S4 Fig) was observed. The paralogous pairs with *Ka/Ks* greater than 1 mostly appeared after the K-Pg boundary, while the paralogous pairs with *Ka/Ks* less than 1 appeared throughout the time window. The mean values of *Ka/Ks* of the combined data from all *Cornus* species also showed an increase without delay after the inferred WGD event (Fig 4B); this trend toward increasing *Ka/Ks* lasted approximately 20 million years.

Tests of the five hypotheses of WGD locations with the 7133 multiple gene groups using WGDgc on the SCG tree revealed that H2 was the best fit and the retention rate of WGD was 0.1, as defined by the probability that the gene copy created at the onset of WGD is not immediately lost (Rabier et al. 2014). H2 also received the largest Akaike weights (S4 Table), and it mapped on the stem of the *Cornus* clade (Fig 1, see red asterisk).

In total, 98 gene groups had the topology showing gene duplication in the ancestor of *Cornus* while meeting the requirement for nodal calibration (S3 Fig). The mixture model analysis of the dates of gene duplication for the ancestral node of *Cornus* (inferred as the WGD event) from these gene trees identified only one major peaks (Fig 4C) with the mean values of 76.10 myr (SD = 2.70) (Fig 4C), slightly older than the estimate using *Ks* (Fig 4A, 74 myr) from pooled data of all *Cornus* species, and within the range identified from the *Ks* data of individual species (66~85 myr).

## Discussion

Ancient whole-genome duplication, or polyploidy, events in plants are often difficult to detect using traditional cytological and genetic approaches. However, the availability of large-scale transcriptome data provides a robust platform to estimate the timing of ancient polyploidy through the analysis of synonymous substitution rates in paralogous genes [10, 14, 21, 22]. Several studies have shown that ancient WGD events in a number of flowering plant lineages are associated with angiosperm diversification [10, 11, 13, 27]. The clustering of polyploidy events around the K-Pg boundary suggests a global impact of the K-Pg event and subsequent changes to climate on plant genome evolution. Evidence for the origination of independent WGDs in many lineages strengthens the inference of its major role in angiosperm evolution [11, 12].

By applying the mixture model analysis to paralogous *Ks* distributions of transcriptomes of eight *Cornus* species, we were able to detect four distinct components that may represent four WGD events (Fig 3). The most ancient components were detected in one species around 150 (*Ks* = 0.99) (Green curves, Fig 3C). Ancient WGD inferred from large *Ks* values could be explained as an artifact of *Ks* saturation in deep phylogeny [26]. Although *Ks* estimates can be used for WGD inferences far beyond the commonly accepted *Ks* threshold of 1, *Ks* saturation effects can cause artificial peaks in deep time [25]. Therefore, we feel that this component might be an artifact caused by the smaller number of longer reads generated from 454 Titanium sequencing of the *C*. *canadensis* transcriptome. The coverage was much lower compared to the data generated for other species (see Table 1).

The second component detected in all species of our study corresponds to a WGD event around 66~85 myr ago (blue line in all *Cornus* species) in separate analyses. The date of this WGD inferred from the *Ks* method (Fig 4A, blue, 74 myr) is very close to the result from gene tree method (Fig 4C, 76.10 myr), suggesting that the *Ks* method with the mix-model is reasonable, when a local species clock for *Ks* is calibrated for conversion of *Ks* values to time. However, it may slightly underestimate the relative timing of WGDs that have been widely reported for seed plants [14]. The timing of this WGD event has been estimated to occur at the very late Cretaceous, and it likely has a close relationship to the WGDs at the K-Pg boundary [14]. The environmental factors responsible for mass extinctions at the K-Pg boundary were thought to have triggered and favored genome duplication events in various angiosperm lineages during this turbulent period of Earth history [70], and here we found evidence in *Cornus*, as well. Mass extinction probably also occurred in Cornales during this period as shown by fossils from the Late Cretaceous through the early Tertiary (Paleocene, Eocene) that represent *Cornus*, *Nyssa*, *Davidia*. *Mastixia*, *Dilpopanax*, and *Hydrangea* as well as some some extinct genera [35, 37–40, 69].

This WGD event maps to the stem of *Cornus* and slightly precedes the divergence of the three major lineages and is older than the age of the oldest fossils for the genus, and associated with the sudden increase of sea surface temperature (Fig 1). Furthermore, our test of the five hypothesized locations for this WGD also suggests a location on the stem of *Cornus* (Fig 1, see red asterisk). These two initial successive divergence events coincide with the origin of the three major lineages of the genus in a remarkably narrow time frame (Fig 1). These results suggest that WGD and the subsequent molecular evolution were likely important forces driving the rapid initial diversification of *Cornus* soon after its origin in the late Cretaceous and that climatic changes in late Cretaceous might have played an vital role in the evolution and diversification of flowering plants.

The third and fourth shared components detected in our analyses correspond to events around 15–23 myr (CCA, CCO, CEL, CFL, CKO, COF) and 7–8 myr (CAL, CCA, CEL, CFL, CKO, COF), respectively (Fig 3; yellow and red curves). These components show obvious high peaks and likely reflect more recent WGD events or recent small-scale duplications (SSDs), such as tandem, proximal, and transposed duplications [71, 72]. Except for *C*. *canadensis*, all species show evidence for at least one of these peaks. This may be again an artifact from the transcriptome of *C*. *canadensis* that had many fewer but longer reads than those of the other species.

### Molecular evolution after WGD

It is widely agreed that polyploidy is a major mechanism of plant evolution and diversification [11]. The origin of novel traits for adaptation to new environments driven by diversifying selection is likely a key for the observed global success of polyploid lineages [61]. Following WGD, genome restructuring and gene functional changes, both closely tied to diversifying selection for new adaptation, are expected to increase the *Ka/Ks* ratio which is enhanced by the relatively small size of the typical initial polyploid population [18, 73]. In our study, we observed an increasing pattern *Ka/Ks* after the late Cretaceous WGD event (Fig 4B). This pattern, if real, might be caused by the environmental changes at the K-Pg boundary through strong selection pressure on the duplicated genomes.

A delay in the increase of the rate of molecular evolution after WGDs is expected if there was a dominant process of gene conversion immediately following WGD [19]. Our results, however, showed no apparent delay of the increase of *Ka* in a 5 myr increment. This suggests either that gene conversion is not a major process in early evolution of duplicated genome in *Cornus* or the 5 myr window is too big to detect the signals from gene conversion that has been hidden by the overwhelming signals from subsequent gene divergence. We believe that the 5myr window is sufficient to conclude that the WGD event was followed by an acceleration of molecular evolution that triggered the rapid divergence of the three extant major lineages of *Cornus* (Figs 1 and 4) and subsequent early cladogenesis within each major lineage [35, 37]. This supports the hypothesis that a genome-wide increase in molecular evolution drives diversification.

### Rate variation of molecular evolution among *Cornus* lineages

Many factors affect the rate of molecular evolution in plants, including natural selection for abiotic and biotic variables (e.g. energy, water availability, temperature, ultraviolet (UV) radiation, species interaction, etc.), generation time, metabolic rate, population size, and mutation rate [74]. In plants, dramatic differences in rates of molecular evolution have been noted between annuals and perennials and between woody and herbaceous species [60]. The differences were assumed to reflect differences in generation time (the time from seed germination to the production of fruits/seeds). In our results, *C*. *canadensis*, the herbaceous, rhizomatous perennial lineage, showed a much longer branch (0.123) than other lineages of the genus (0.094–0.097, Fig 1 and Table 1), similar to findings of previous phylogenetic studies using several plastid and nuclear genes [35, 69, 75–77]. Xiang et al. [35] hypothesized that genome-wide acceleration of molecular evolution might have occurred in the herbaceous lineage as a consequence of harsh environmental conditions at high latitudes and altitudes (circumboreal areas) of its distribution. Our results from analyses of transcriptome data also indicate that the synonymous substitution rate (*Ks*) of *C*. *canadensis* is greater than that of the other *Cornus* species when calculated using the outgroup references *Alangium* (0.543 vs 0.4334–0.461) and *Dichroa* (0.6133 vs 0.5051–0.5277) (Fig 2). Paralogous *Ks* for *C*. *canadensis* also has the greatest maximum value within the 200-myr timeframe (1.32) and the fastest molecular clock (*C*_*Ks*_ = 0.00329 substitutions per site per year; Table 2). Despite the biases of 454 sequencing of *C*. *canadensis*, the method of sequencing had low impact on the *Ks* of orthologous gene copies between species of *Cornus* and outgroups. These data support the hypothesis of a genome-wide acceleration of molecular evolution in the only herbaceous lineage of *Cornus*. Natural selection may act on particular genes, while population size and mutation rate affect the entire genome. We hypothesize that an accelerated rate of molecular evolution in *C*. *canadensis* may have resulted from a combination of factors that potentially enhance nucleotide substitution rate, including a likely shorter generation time, strong selection on adaptive traits to grow in harsh environments (e.g. the reduction to an annual above-ground stem, explosive pollen release; see Edwards et al. [78]), smaller effective population sizes due to scarcity of pollinators, and a possible increased mutation rate in boreal habitats where UV light is strong and summer day-length is long.

### Correlation of WGDs with dramatic temperature changes

The establishment of polyploids is believed to be more likely during periods of environmental stress because they have greater genetic flexibility and phenotypic plasticity [14, 79, 80]. A recent study of *Brachypodium distachyon* found that the distribution of diploid and tetraploid genotypes is associated with aridity and annual precipitation gradients, with polyploid populations more prevalent in dry environments [81]. We also explored the relationship between the variation of reconstructed geologic temperature through time and WGDs. The data suggest that the occurrence of an ancient, major WGD in *Cornus* coincides with an increase in the sea surface temperature [82] during the late Cretaceous (Fig 1). Climate change and environmental stress are known to increase the frequency of unreduced gametes (diplogametes; e.g. Kurschner et al. [83]). Heat stress in *Rosa* species and cold stress in *Arabidopsis thaliana* lead to increased unreduced gamete formation through alterations in spindle formation during meiosis II [84] and in post-meiotic cell plate formation and cell wall establishment [85].

The ancient dogwood genome duplication likely occurred through a neutral mechanism related to increased unreduced gamete formation during the K-Pg period. The WGD was evolutionarily conserved, which was potentially linked to the changed climate with increased sea surface temperature around the K-Pg boundary and shortly thereafter.

## Conclusions

Analyses of the synonymous substitution rate in the transcriptomes of eight species of *Cornus* revealed a putatively common ancient WGD event at the K-Pg boundary. The tempo of this event was correlated with the timing of the initial diversification of *Cornus*, as well as an abrupt increase of the sea surface temperature. The evidence is consistent with the hypothesis that environmental stress in the past promoted genome doubling and the formation and survival of polyploids. We also find that the molecular evolution rates were especially high in the transcriptome of *C*. *canadensis*, an herbaceous species that inhabits the harsh environmental conditions of the Arctic region.

## Supporting information

S1 Fig The customized pipeline of software and methods applied in our paper. (TIF)

S2 Fig The five hypothesis on the possible locations of WDGs on the SCG phylogeny within the time window of 66–85 myr. H1: WGD on the stem of the *Cornus-Alangium* clade; H2: WGD on the stem of the *Cornus* clade; H3: independent WGDs on the stem of the *Cornus* clade and on the stem of *Alangium*, respectively; H4: independent WGDs on the stem of the *Cornus* BW group (represented by CAL and CCO) and on the stem of the remainder of the genus (represented by CCA, CEL, CKO, CFL, CCN and COF, respectively; and H5: independent WGDs on the stem of *Alangium*, on the stem of the *Cornus* BW group, and on the stem of the remainder of the genus.
(TIF)

S3 Fig (a) The criteria used for gene trees indicating gene duplication in the common ancestor of *Cornus*. The tree should contain *Alangium* and two subclades of *Cornus*, consisting of any two or more species that represent the deepest phylogenetic divergence within *Cornus*. The divergence times of the two paralogous clades in these trees were estimated under the assumption of a relaxed molecular clock using R8S. We used the late Cretaceous fossil (minimum age of 68 myr) of CC group for the node departing the BW species, and the maximum age of 86.28 myr for the node uniting *Cornus* and *Alangium* in our estimation. The detailed information of nodes with red circles were represented in (b). The nodes with blue circles (if available) in each gene tree were constrained using fossils (S1 Table).
(TIF)

S4 Fig The distribution *Ka/Ks* values of paralogous pairs through time from 200 myr to present (x-axis) for (a) *C*. *alternifolia*, (b) *C*. *capitata*, (c) *C*. *canadensis*, (d) *C*. *controversa*, (e) *C*. *ellipica*, (f) *C*. *florida*, (g) *C*. *kousa*, (h) *C*. *officinallis*. The values of *Ka/Ks* (y-axis) are indicated by open dots. The vertical green, blue, yellow and red dashed lines mark absolute ages of corresponding normal components of paralogous *Ks* shown in Fig 3. The horizontal black dashed lines indicate the *Ka/Ks* value of 0.5 and 1.
(TIF)

S1 Table The records of fossils, settings and references used in divergence time analyses. (XLSX)

S2 Table The BIC value of each number of groups (NG) for each species of *Cornus*. (XLSX)

S3 Table Detailed information for each normal distribution groups for eight *Cornus* species and combined data. (XLSX)

S4 Table The summaries of five hypotheses testing using WGDgc. (XLSX)
