# Supplementary figures and images for: Whole-genome duplication and molecular evolution in Cornus L. (Cornaceae) – Insights from transcriptome sequences
Source: PLoS One. 2017 Feb 22;12(2):e0171361. doi: 10.1371/journal.pone.0171361 (PMC5321274; doi:10.1371/journal.pone.0171361)

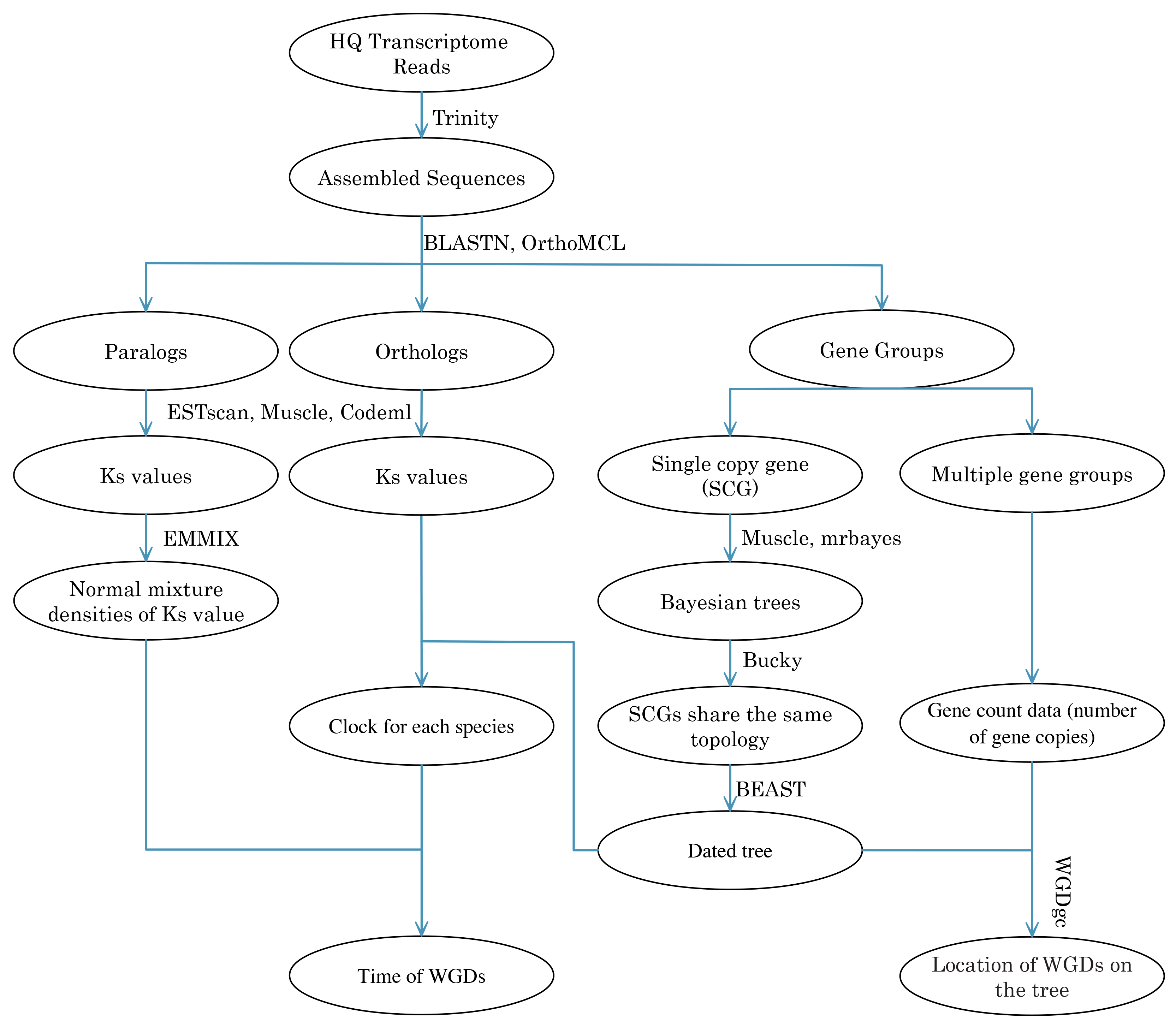

Supplement: S1 Fig — (TIF) [file pone.0171361.s001.tif]

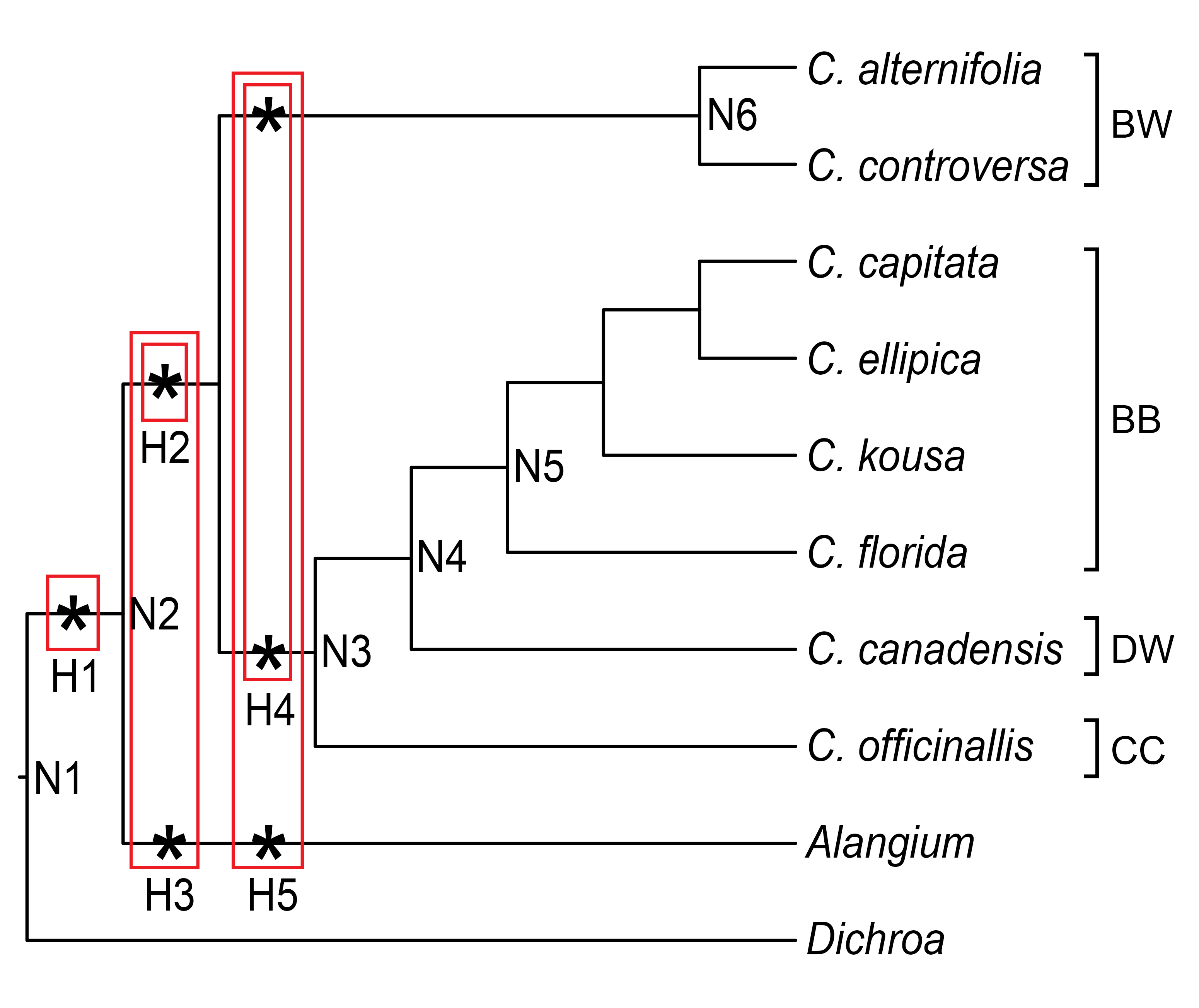

Supplement: S2 Fig — H1: WGD on the stem of the Cornus-Alangium clade; H2: WGD on the stem of the Cornus clade; H3: independent WGDs on the stem of the Cornus clade and on the stem of Alangium, respectively; H4: independent WGDs on the stem of the Cornus BW group (represented by CAL and CCO) and on the stem of the remainder of the genus (represented by CCA, CEL, CKO, CFL, CCN and COF, respectively; and H5: independent WGDs on the stem of Alangium, on the stem of the Cornus BW group, and on the stem of the remainder of the genus. (TIF) [file pone.0171361.s002.tif]

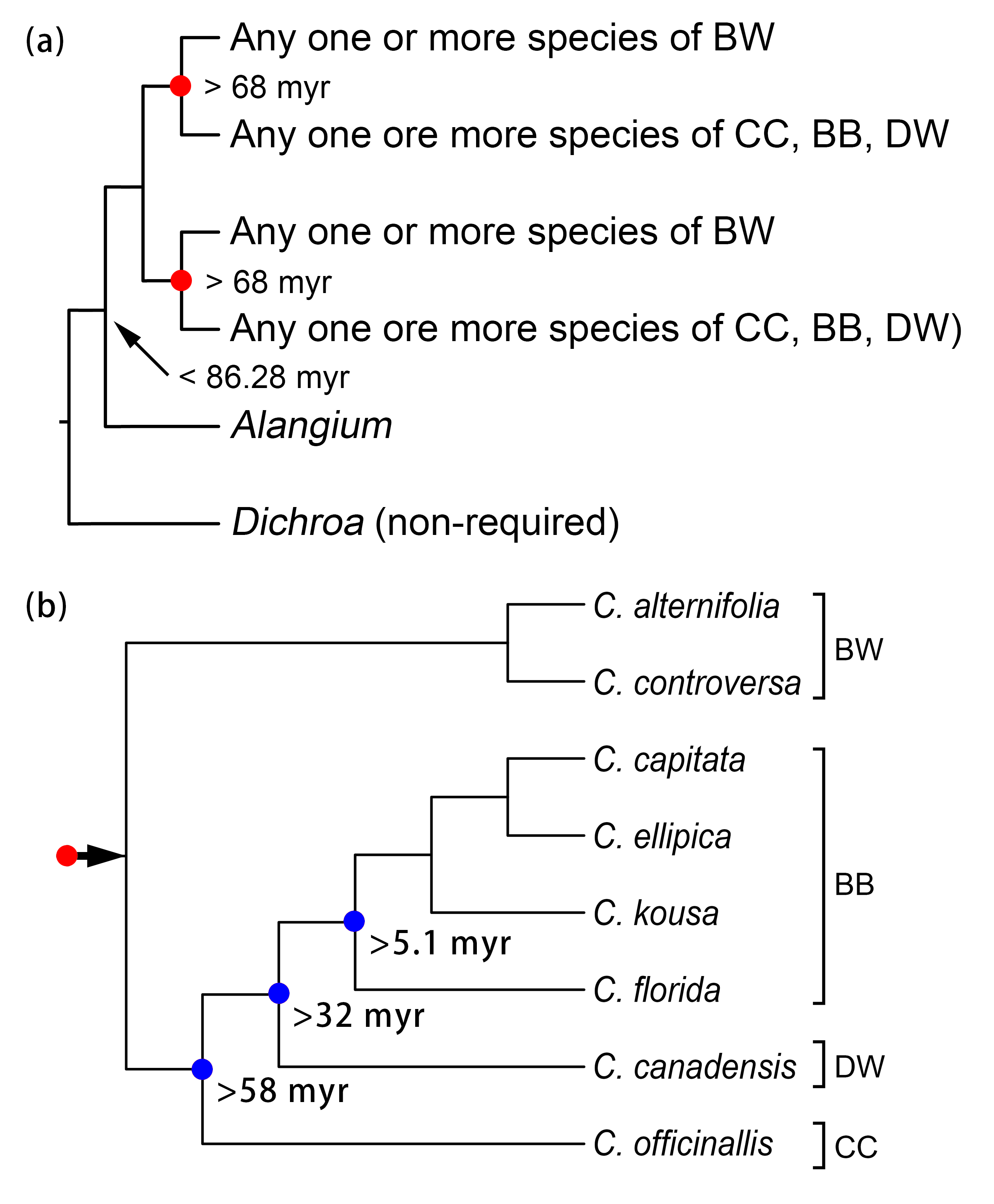

Supplement: S3 Fig — (a) The criteria used for gene trees indicating gene duplication in the common ancestor of Cornus. The tree should contain Alangium and two subclades of Cornus, consisting of any two or more species that represent the deepest phylogenetic divergence within Cornus. The divergence times of the two paralogous clades in these trees were estimated under the assumption of a relaxed molecular clock using R8S. We used the late Cretaceous fossil (minimum age of 68 myr) of CC group for the node departing the BW species, and the maximum age of 86.28 myr for the node uniting Cornus and Alangium in our estimation. The detailed information of nodes with red circles were represented in (b). The nodes with blue circles (if available) in each gene tree were constrained using fossils (S1 Table). (TIF) [file pone.0171361.s003.tif]

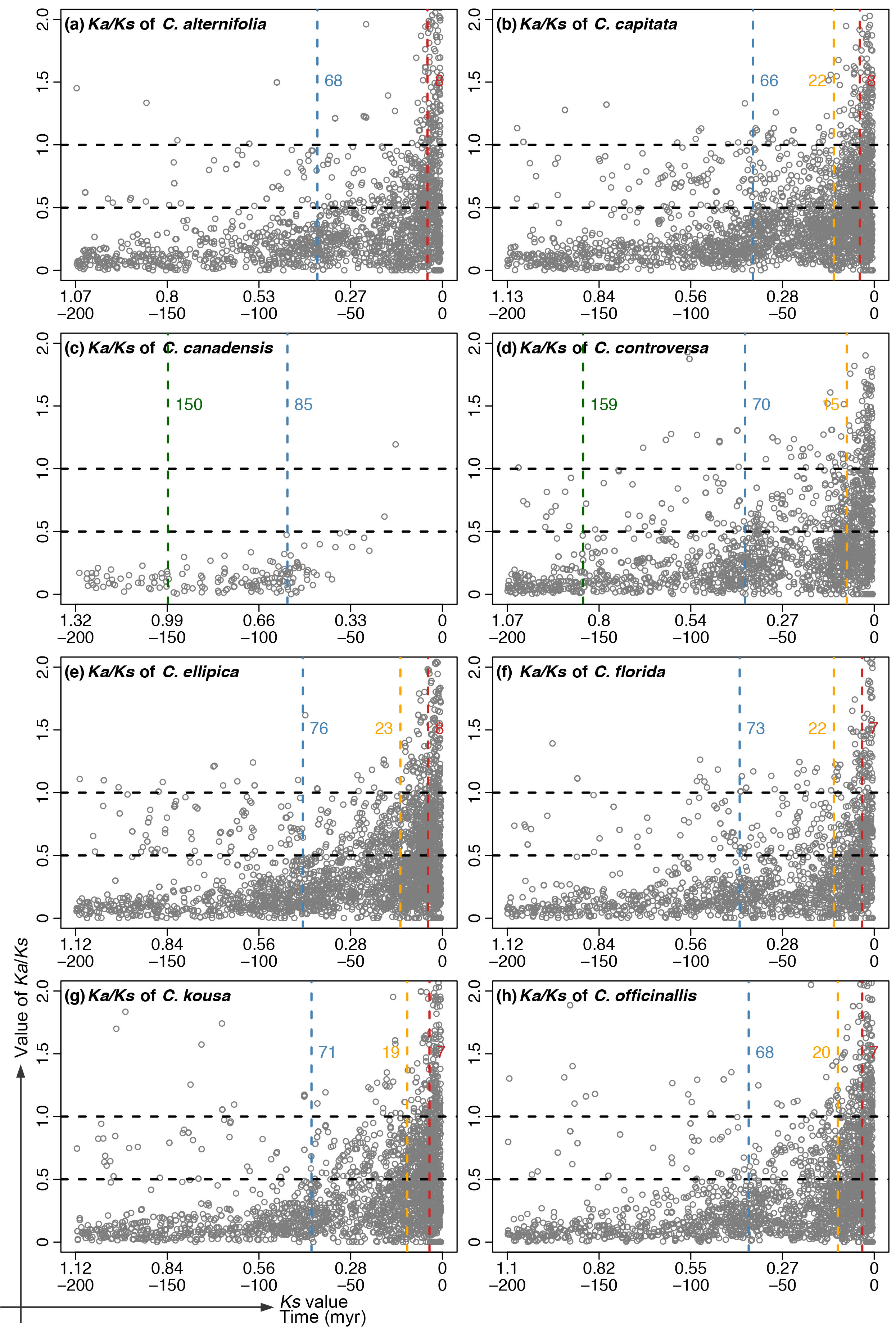

Supplement: S4 Fig — The distribution Ka/Ks values of paralogous pairs through time from 200 myr to present (x-axis) for (a) C. alternifolia, (b) C. capitata, (c) C. canadensis, (d) C. controversa, (e) C. ellipica, (f) C. florida, (g) C. kousa, (h) C. officinallis. The values of Ka/Ks (y-axis) are indicated by open dots. The vertical green, blue, yellow and red dashed lines mark absolute ages of corresponding normal components of paralogous Ks shown in Fig 3. The horizontal black dashed lines indicate the Ka/Ks value of 0.5 and 1. (TIF) [file pone.0171361.s004.tif]
